# Occurrence and Control of *Legionella* in Recycled Water Systems

**DOI:** 10.3390/pathogens4030470

**Published:** 2015-07-01

**Authors:** Patrick K. Jjemba, William Johnson, Zia Bukhari, Mark W. LeChevallier

**Affiliations:** 1 American Water Research Laboratory, 213 Carriage Lane, Delran, NJ 08075, USA; E-Mail: william.johnson2@amwater.com; 2 American Water, 1025 Laurel Oak Road, Voorhees, NJ 08043, USA; E-Mails: zia.bukhari@amwater.com (Z.B.); mark.lechevallier@amwater.com (M.W.L.)

**Keywords:** amoeba, disinfection, Legionella, protozoa

## Abstract

*Legionella pneumophila* is on the United States Environmental Protection Agency (USEPA) Candidate Contaminant list (CCL) as an important pathogen. It is commonly encountered in recycled water and is typically associated with amoeba, notably *Naegleria fowleri* (also on the CCL) and *Acanthamoeba* sp. No legionellosis outbreak has been linked to recycled water and it is important for the industry to proactively keep things that way. A review was conducted examine the occurrence of Legionella and its protozoa symbionts in recycled water with the aim of developing a risk management strategy. The review considered the intricate ecological relationships between Legionella and protozoa, methods for detecting both symbionts, and the efficacy of various disinfectants.

## 1. Introduction

Recycled water is water, which, as a result of treatment of waste, is suitable for direct beneficial purposes. Water recycling is a very ancient practice. However, the practice has intensified amid tremendous advances in water treatment technologies, an increased interest in resource conservation, enhanced water demand stress, drought episodes and the associated debate about climate change. *Legionella pneumophila* has a high epidemiological significance and is currently on the candidate contaminant list 3 (CCL3; [1]). It was one of 20 priority organisms of concern in recycled water quality identified recently [2]. Several other research groups have also recognized the importance of *Legionella* sp. in recycled water [3,4,5]. Although no legionellosis outbreak has been directly associated with recycled water, outbreaks related to water intrusion [6,7], cooling towers [8], a mist humidifier [9], and distribution systems [10] have been reported. Thus, it is in the recycled water industry’s best interest to proactively prevent future outbreaks as *Legionella* spp. are very prevalent in the product. Overall, Legionella infections occur sporadically and in outbreaks but in most instances, the source of infection is not always easily deciphered. Currently, legionellosis is the most common waterborne disease reported in the US and surveillance data show a steady increase of cases [11,12]. Members of the genus are Gram-negative bacteria, which occur ubiquitously in aquatic and engineered systems. To date, 52 species and 70 serotypes have been identified [13,14]. Although fairly ubiquitous, *Legionella* spp. thrive in warm (25–42 °C) water, particularly in areas where water stagnates [15]. They occur as planktonic cells, biofilm denizens, or as intracellular symbionts in protozoa, especially free-living amoeba.

Models to understand the risk from *Legionella* sp. in potable water systems have been developed. There are apparent differences between recycled and potable water. Most distinct is the relatively higher level of nutrients in recycled compared to potable water. Despite differences, modeling processes from potable systems can be a useful basis for comparative analysis in managing risk from *Legionella* sp. in recycled water systems. Occurrence of *Legionella* sp. in potable water plumbing systems has been documented by numerous research groups (Table 1). Evidence from potable water systems showed most abundance of *Legionella* spp in heated units, such as cooling towers, hot tubs, and hot water tanks. Some of the key considerations in potable water systems that warrant inclusion in a risk assessment and management strategy are the importance of temperature and protozoa host symbionts. For example, *L. pneumophila* increased by three log units in cooling tower foam compared to the bulk water column [16]. Foaming is a common occurrence in wastewater treatment especially where organic acids and other antiscalants are used. Colburne *et al.* [16] recommended treatment processes which minimize foaming, during wastewater treatment processes for generating recycled water. Dead end points, with long retention time and areas in the distribution system receiving water flow from more than one direction were more prone to colonization by *Legionella* spp. [17].

**Figure 1:**
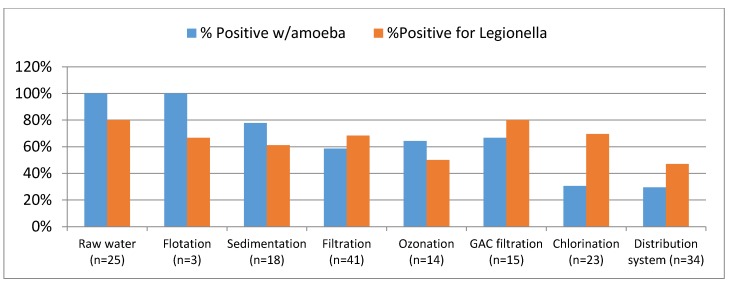
Positive samples for amoeba and *Legionella* spp. in ten treatment systems (Figure compiled from Loret and Greub [18]).

Some amoeba species internalize bacteria. Data from Table 1 also strongly indicate the need to consider protozoa in addressing the impact and risks associated with *Legionella* sp. in water systems. Although conducted in potable water systems, Figure 1 shows the intricate association of amoeba with *Legionella* sp. throughout the treatment process and subsequent distribution. Both amoeba and Legionella in those instances were detected using conventional culture methods and polymerase chain reaction (PCR), but the latter were rarely detected by conventional culture methods [18]. The results also show the replication of *Legionella* spp. in granular activated carbon (GAC) filters and survival after disinfection, continuing to grow in distribution systems.

Protozoa were also consistently detected in other Legionella-infested assemblies. For example, protozoa were detected in 29% (*n* = 231) of hot water recirculation systems. A majority (93%) of the protozoa detected were amoeba [19]. Flagellates and ciliates were detected in only 26.8% and 3.6%, respectively. Similarly, *Acanthamoeba* sp. and *Vermamoeba vermiformis* were detected in two potable water distribution systems using molecular probes [20]. Yamamoto *et al.* [21] detected ciliates and flagellates in cooling towers.

**Table 1:** Selected examples of *Legionella* spp. occurrence in potable water distribution and plumbing systems.

Location	Organism	% Occurrence	Mean Density in Positive Samples	Refs.
Blacksburg-Christiansburg-VPI Water Authority (Virginia, USA; *n* = 29)	*Legionella* spp.	69	187 gc/mL	[20]
	*L. pneumophila*	13.7	9.8 gc/mL	
	*Vermamoeba vermiformis*	27.6	1.2 ×10^4^ gc/mL	
	*Acanthamoeba* sp.	13.7	2.2 gc/mL	
Pinellas County, FL (USA; *n* = 15)	*Legionella* spp.	100	100.8 gc/mL	[20]
	*L. pneumophila*	20	90.4 gc/mL	
	*Vermamoeba vermiformis*	73.3	781.7 gc/mL	
	*Acanthamoeba* sp.	6.7	ND *	
Catalonia (Spain)	*Legionella* spp.	35	6.9 CFU/100 mL	[19]
Nara/Gifu/Aichi/Shizuoka (Japan)	*Legionella* sp.		1.6 CFU/100 mL	[21]
	Amoeba		2.4 MPN/100 mL	
	Ciliates		1.1 MPN/100 mL	
	Flagellates		2.8 MPN/100 mL	
Pittsburg Psychiatric Hospital	*Legionella* sp.	50%–90%	117.5/swab	[22]
London (cooling tower foam)	*L. pneumophila* 1		10^5^ cfu/mL	[16]
Miyazaki, Japan (spa)	*Legionella* sp.		1.5 × 10^7^ cfu/L	[23]
Shizuoka, Japan (outdoor thermal spring)	*Legionella* sp.		5.7 × 10^5^ cfu/L	
Shizuoka, Japan (indoor thermal spring)	*Legionella* sp.		8.8 × 10^5^ cfu/L	
Singapore	*Legionella* sp.	15.6% (cooling towers); 12.4% (fountains)	No reported	[14]
Israel	*Legionella* spp.	7.2–18.2 over 5 years	Not reported	[13]

* ND = Not detected (but some gene copies detected in the biofilm).

The risk and infectivity of Legionella can differ depending on the source water, treatment processes, and intended use of the recycled water. A recent survey of 10 recycled water systems highlighted typical uses in the United States (Table 2). Also included in the table is the potential for each use to generate aerosols. Legionella is a nonconventional waterborne pathogen, as it is not transmitted orally. Transmission is through mechanical means, which generate aerosols including sprinklers, cooling towers (air-conditioning) and shower heads; mechanisms which prominently feature in the use of recycled water. Once inhaled in aerosols, the bacteria are internalized in the lungs by alveolar macrophages and epithelial cells, replicate within the phagosomes and eventually lyse the host macrophages. This process is similar but not identical to the organism being parasitized by protozoa [24]. The ecological relationship between Legionella and protozoa is reviewed underneath.

**Table 2:** Typical uses of recycled water for 10 systems in the US and related potential to generate aerosols.

Use	System (%) ^1^	Potential for Generating Aerosols
Irrigation (parks, medians, farms, lawns, *etc.*)	90	Low (drip) to high (aerial spray)
Cooling towers/Boilers	50	High
Construction	20	Moderate
Dust control	10	Moderate
Washing (cars, windows)	10	Moderate
Street sweeping	10	Moderate
Fire fighting	10	Moderate
Toilet/Urinal flushing	30	Low
Groundwater recharge	20	Low
Animal watering	10	Low
Wetlands	10	Low

^1^ Total is more than 100% as most systems utilized recycled water for multiple uses. Source: Table compiled from [25].

## 2. Ecology of *Legionella* sp. and Its Protozoa Host

Ecology is the study of the distribution, activities and interactions of organisms with their habitats. Such studies normally entail the isolation, identification and measurement of the activities of the organisms, assessment of their interactions with other organisms, and determining their response to abiotic environments [26]. A typical recycled water distribution system is inherently prone to intermittent flow changes as a result of changes in water pressure and demand [27]. It also tends to have low disinfectant residual and relatively high levels of nutrients (e.g., organic carbon, nitrogen and phosphorus), which in turn support growth and survival of microorganism including *Legionella* sp. [25,28]. These characteristics create an environment with many dead ends and even more dissipation of the disinfectant residual, an important preservative.

*Legionella* sp. can multiply in biofilms and/or as an intracellular symbiont with protozoa in the distribution system [18]. Biofilms are assemblages of bacteria encased in extracellular polymeric compounds, attached to phase boundaries or surfaces. Such an adherent and hydrated environment protects bacteria from desiccation and harmful chemicals [29].

Colonization of biofilms by *Legionella* spp. occurred within a short timeframe attaching to the substratum using pili and flagella [30]. Expression of the *flaA* gene (involved in *L. pneumophila* flagellum assembly and movement to the biofilm) also increased by 40% under a biofilm environment. However, *L. pneumophila* did not show an absolute requirement for pili and secretions implicated in the attachment and retention in biofilms [24]. Colonization and retention of pili deficient mutants was sustained in the presence of amoeba. *Legionella pneumophila* also expressed competence and adherence-associated pili (CAP) on its surface, which enhanced its ability to adhere to surfaces and biofilms [31]. Under intercellular and biofilm environments, *Legionella* sp. can be protected from disinfectants [32,33], with important ecological ramifications highlighted later in the review.

**Figure 2:**
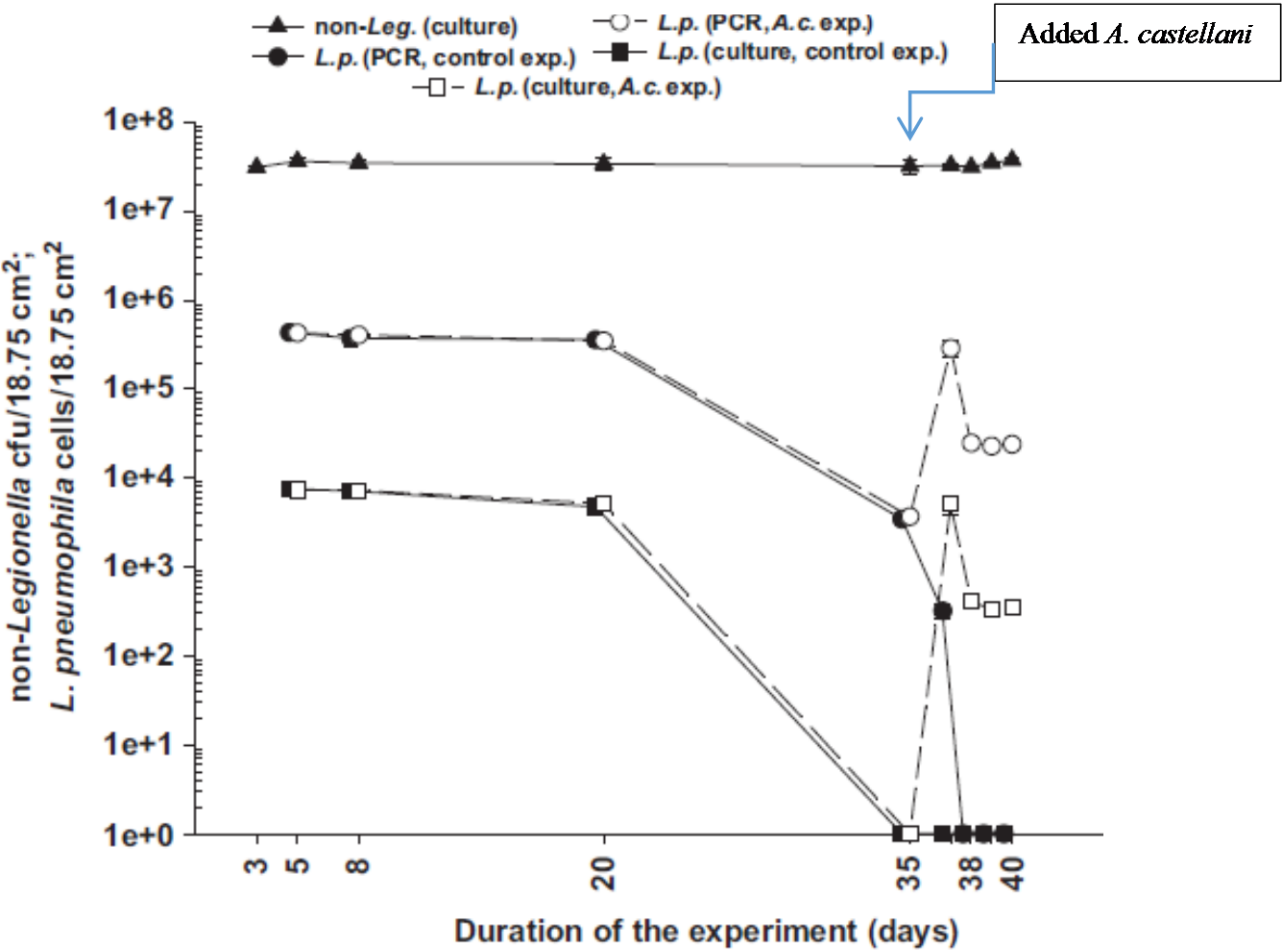
Evolution of biofilm-associated Legionella and a mixture of other (non-Legionella) bacterial species. *Acanthamoeba castellani* (A.c.) were added in the reactor on day 35. Non-Legionella bacteria in the reactor included *Aeromonas hydrophila*, *Escherichia coli*, *Flavobacterium breve* and *Pseudomonas aeruginosa*. (Source: [30]).

Coordination between *Legionella* sp. and biofilm colonization was displayed by a dramatic decrease of biofilm-associated *Legionella* sp. in a rotating annular reactor (RAR) in the absence of amoeba (Figure 2). By contrast, addition of *Acanthamoeba castellani* reversed the trend and increased the density of Legionella by 2.9 log units in the biofilm. Amoebae were lysed within 72 h, releasing more *Legionella* sp. into the bulk water. Those results collectively demonstrated enhanced survival of *Legionella* sp. in the presence of the amoeba host. From a practical perspective, control of *Legionella* sp. in recycled water may not be feasible unless it is combined with controlling amoebae as well.

Amoeba exist in a vegetative form (trophozoites) or in a resting form (cysts), the latter enabling survival under adverse environmental conditions including low nutrients and higher temperatures. After ingestion of bacteria, amoebae formed small vesicles of <5 μm which harbor *Legionella* and other symbionts such as *Mycobacterium*, *Enterobacterium* and *Vibrio* spp [34]. Each vesicle contained an estimated 20 to 200 bacteria. Feeding experiments significantly increased the number of vesicles formed by *A. castellani* and *A. polyphaga* when the amoeba were fed on a mixture of *E. coli* and *Legionella* sp. compared to amoeba solely fed on either bacterial species alone [34]. There was no evidence of preferential feeding of the amoeba on either type of bacteria in the mixture. Concrete enumeration of each bacterial type in the tightly packed vesicles was not feasible.

Some *Legionella* species, notably *L. drancourtii* and *L. jeonii*, are obligate intracellular denizens of protozoa, unable to grow axenically in free media without protozoa [35]. The internalized bacteria are able to grow within vesicles) and even survive in amoeba cysts. These internalization and release processes may contribute to survival in a viable but non-culturable (VBNC) state [36]. When environmental conditions become suitable for encystation, *Legionella* sp. egress and re-infect new amoebae. Through this behavior, free-living amoeba share many common features with mammalian phagocyts such as macrophages, enhancing Legionella’s ability to resist phagocytosis [37,38]. Thus, amoeba may act as Trojan horses, providing a training ground for *Legionella* sp. to enhance its infectivity and pathogenicity to humans with important ecological and epidemiological implications.

Thomas *et al.* [39] analyzed free-living amoeba at each step of a water treatment system and recovered bacteria-infected amoeba mainly from filter media. They attributed the increased presence of infected amoeba in this environment to biofilms, which favored contact between the free living amoeba and bacteria. Higher densities of bacteria-infected amoeba in biofilms compared to the surrounding water in cooling towers were also documented by Berk *et al.* [40]. Biofilms are initiated by attachment and adherence of organisms to one another and/or to abiotic surfaces. Composition of the surface material plays a major role in determining the extent of adherence. For example, *L. pneumophila* attached quite well to plastic and other materials commonly used in recycled water piping, reservoirs and appurtenances [34]. Attachment, colonization and subsequent formation of the biofilm was also enhanced by carbon, especially at relatively low temperatures (*i.e.*, 20 °C; [41]).

**Figure 3:**
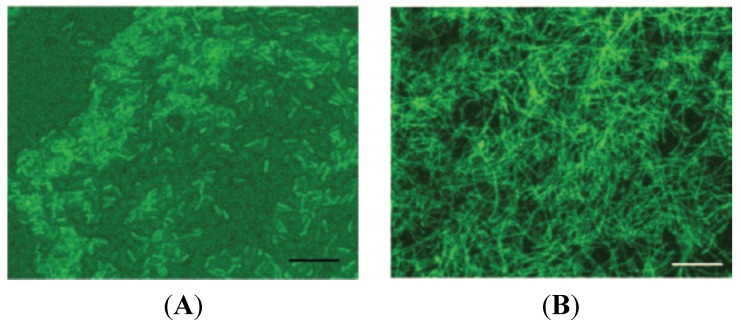
Structure of *Legionella pneumophila* biofilm grown at (**A**) 25 °C for 18 days [non-filamentous growth] and (**B**) 37 °C for 6 days (filamentous growth). Bars are 10 μm. Figure adapted from [29] with permission from American Society for Microbiology.

*Legionella pneumophila* biofilms were significantly influenced by temperature and emerged within three days at 37 °C and 42 °C compared to 11 days at 25 °C [29]. The biofilms formed at 25 °C were more adherent, thinner and rod-shaped but non-filamentous whereas those formed at 37 °C and 42 °C were filamentous (Figure 3). The filamentous biofilms were possibly a fitness trait against adverse environments [33]. These findings can have significant ecological implications for public health where the recycled water is subjected to elevated temperatures typical of water heaters and cooling towers. At elevated temperatures the formed biofilms will be less stable and more prone to slough off releasing *Legionella* spp. (and possibly associated protozoa) into the bulk water. The released organisms ultimately end up in aerosols. Because of the importance of temperature extremes on *Legionella* sp. and amoeba ecology, their occurrence in cooling towers and water heaters is briefly reviewed underneath.

### 2.1. Cooling Towers

Cooling towers operate through evaporation of water into the atmosphere. The use of recycled water for cooling towers in the United States and other countries is increasing. Yamamoto *et al.* [21] surveyed 40 cooling towers in Japan where water temperatures ranged between 8.3 °C to 35 °C and detected *Legionella* sp. in 73% of the towers. Of the 359 isolates identified, 90% were *L. pneumophila*, with serogroup 1 as the most prevalent. Other serogroups included 3 (18 strains), 4 (2 strains), 5 (8 strains), 6 (26 strains) and unidentified serogroups (65 strains). Maximum Legionella densities were detected in water of pH 8.4 to 9.1 and temperature 26.3 °C to 29.9 °C [21]. No water of acidic pH was encountered in the towers. Predominantly alkaline conditions in cooling were also reported by Miller and Simpson [42]. The density of *Legionella* sp. in the towers correlated positively with temperature, water pH, and amoeba abundance; but not with heterotrophic bacteria (Table 3). A similar conclusion about HPCs was reached by Serrano-Suárez *et al.* [19]. The highest Legionella densities of 10^5^ CFU *Legionella* sp./100 mL in the towers registered by Yamamoto *et al.* [21] were in summer but *Legionella* sp. were also consistently present in the towers at 10^2^ to 10^3^CFU/mL in winter. Protozoa (*i.e.*, amoeba, ciliates and flagellates) of several taxa were detected in 98% of the samples throughout the year at densities of 10 to 10^3^ MPN/100 mL. The towers contained biofilms and deposits, which, together with the protozoa, may have protected *Legionella* sp.

**Table 3:** Correlation of various parameters in 40 cooling towers in Japan.

Parameter	Water Temperature	pH	Heterotrophic Bacteria	Legionella
Heterotrophic bacteria	0.190	0.331 *		
Legionella	0.311*	0.319 *	0.104	
Ciliates	0.332*	0.388 *	0.146	0.300 *
Flagellates	0.122	0.042	−0.079	0.383 *
Amoeba	0.328*	0.208	0.002	0.300 *

* Correlation was significant (*p* < 0.01; 99% confidence; Source: [21].

More recently Mouchtouri *et al.* [43] detected *Legionella* sp. in 49% of 96 cooling towers surveyed in Greece. One third of the samples tested had ≥40 cfu/L and of the 69 isolates, 80% were *L. pneumophila*, with 75% as *L. pneumophila* serotype 1. A positive correlation was found in towers with <0.5 mg residual chlorine/L. *Legionella* spp. were also detected in several cooling towers in Florida [17] and in 47% of the cooling towers sampled in Singapore [14]. *Legionella* sp. occurrence in recycled water-cooling towers has not been deliberately studied but countries such as Singapore have heavy usage of recycled water.

### 2.2. Water Heaters

Although recycled water is not yet widely used for domestic purposes, it is used at some commercial facilities for boiler makeup water and other industrial processes where heating is routinely conducted. Parallels can be drawn from experiences with occurrence of *Legionella* spp. and protozoa in domestic hot water systems. Serrano-Suárez *et al.* [19] reported most of the Legionella isolates in hot water circulation systems between 25 °C and 45 °C but some isolates were obtained from locations outside this temperature range. A few *Legionella* spp. survived at 70 °C [32]. Protozoa host proliferation and survival at high temperatures for thermophiles such as *Vermamoeba vermiformis*, has been documented although prevalence of amoeba was significantly lowered above 60 °C [44,45]. *Acanthamoeba polyphaga* cysts were resistant to 62 °C and although reduced by 5 logs after a 2 h contact of 62–65 °C, viable cysts were not totally eliminated until heating at 70 °C for at least 30 min [18]. Similarly *Vermamoeba vermiformis* cysts persisted until contact to 60 °C for 30 min. These observations have ramifications for persistence of free-living amoeba and symbiotic *Legionella* sp. in water heaters and boilers. Berk *et al.* [34] reported lysis of amoeba at 35 °C, releasing the bacteria loaded vesicles. The released bacteria were still viable.

## 3. Challenges in Detecting *Legionella* spp. and Their Protozoa Hosts

*Legionella* spp. were initially recovered using guinea pigs and embryonated hen eggs [46]. However, that process was very expensive and time consuming. Five modern laboratory methods for detecting *Legionella* spp. in recycled water and about an equal number of methods for detecting *Acanthamoeba* sp. and *Naegleria* sp. (two more of the 20 priority organism of importance in recycled water previously highlighted (Table 4) were used by different laboratories. Most of them involved isolation of the respective organism using a formulated media. However, most of them had not been validated through a round robin testing process [2]. The validation process utilizes statistically sound testing, to identify sensitive, specific, and reproducible methods that help to improve the reliability of monitoring programs [47]. Validation also establishes the operational limits and laboratory performance specifications relevant to the intended use of the method. If conducted properly, validation should address sampling and sample preservation issues; include analytical blanks, reference standard samples, spikes and recoveries from such spikes, duplicate sample, and calibration checks; establish method detection limits and method performance range; include positive and negative controls and sterility checks; assess viability and infectivity status of the organism; establish sources of interferences that can affect data reliability; examine variable matrix applicability (e.g., varying pH, conductivity, and organic carbon levels); and exert consistent total quality management as to ensure high specificity, sensitivity, precision, and accuracy.

Data based on these less than perfect methods reveal that the density of *Legionella* sp. may greatly vary in response to the density and composition of protozoa and biofilms in the system. Where *Legionella* spp. get embedded in protozoa, they remain protected and adapt a wide range of forms including the viable but non-culturable. For example, *Legionella* spp. were detected by PCR in 41% of 231 samples from hot water recirculation systems but a culture-based method detected the organism in only 27% of the samples [19]. Logistic analysis associated *Legionella* sp. with at least 0.095 mg Fe/L. Iron is essential for the growth of *Legionella* sp. whereas copper is inhibitory. Both metals can be derived from pipes and appurtenances, depending on composition of the distribution system infrastructure.

**Table 4:** Summary of Methods for Detecting Legionella and Two Important Amoebae in Reclaimed Water.

Organism	Detection Method ^a^	Aggregate Score	Round-Robin Tested?	Growth Media	Key Refs.
*Acanthamoeba*	Membrane filtration and incubation	29	No	Non-nutrient agar and peptone yeast extract glucose (PYG)	[48]
	**Membrane filtration, enrichment on Neff's media and PCR**	**31**	**No**	Neff's saline non nutrient agar and peptone yeast extract glucose (PYG)	[49,50,51]
	Centrifugation and incubation	29	Yes	Non-nutrient agar	[52,53]
	**Membrane filtration, plaque formation and PCR**	**30**	**No**	Non-nutrient agar	[54]
*Naegleria*	Membrane filtration (or centrifugation) and incubation	31	No	Non-nutrient agar and peptone yeast extract glucose (PYG)	[55]
	Membrane filtration, enrichment, and PCR	34	No	Neff's saline non-nutrient agar with *E. coli* lawn	[55]
	Membrane filtration, plaque formation, and PCR	32	No	Non-nutrient agar	[54]
	Enzyme-linked immunosorbent assay (ELISA)	38	No	Non-nutrient agar	[56]
	Isoenzyme electrophoretic focusing (IEF)	32	No	Non-nutrient agar	[56,57]
	Restriction fragment length polymorphism (RFLP)	32	No	Non-nutrient agar	[56,58]
	**Concentration by centrifugation and culture**	**39**	**No**	Non-nutrient agar	[59,60]
	**Real-time PCR**	**40**	**No**	Not applicable	[61,62,63]
	Concentration by centrifugation and then nested PCR	30	No	Nutrient agar	[64,65]
*Legionella*	MF, heating, acidification and plating on CYE	37	No	CYE agar	[66]
	MF, acidification and plating on BCYE	37	No	BCYE agar	[28,67]
	Direct fluorescent-antibody (DFA) staining	36	No	Not applicable	[67]
	**PCR with EnviroAmp kit**	**47**	**No**	Not applicable	[67,68]
	**Semi-nested PCR**	**41**	**No**	Not applicable	[69]

^a^ Detection method in bold were most comprehensive based on an aggregate scoring exercise (see details in [2]).

In all instances of the methods listed in Table 4, the use of PCR to detect either *Legionella* sp. or its protozoa host was more sensitive than conventional culture methods. Similar observations were made by Mario *et al.* [70] and Merault *et al.* [71]. PCR-based methods also had a much shorter turnaround time (*i.e.*, hours instead of days to weeks). However, it has been urged that presence of nucleic acids and the resultant amplification by PCR in itself has no reflection on whether the detected nucleic acid material is from live or dead cells or how infective the cells are. To detect viable cells using PCR-based methods, modifications incorporated propidium monoazide (PMA) or ethidium monoazide (EMA) dyes with bacteria [72,73] and protozoa [74]. Both dyes preferentially penetrate dead or damaged cells, but not viable cells with intact cell membranes. Once inside the cell, the dye molecules intercalate with DNA and covalently bind upon exposure to light. The photoactive moiety forms a stable DNA-PMA or DNA-EMA complex that interferes with PCR amplification. Thus, when applied, only DNA from viable cells (e.g., those with intact membranes) is amplified during PCR, enabling differentiation between viable and nonviable cells. However, the process can succumb to interference from the matrix. For example, Gedalanga and Olson [75] used this technique on raw sewage and chlorine-disinfected recycled water effluents and found no distinction between amplification of live and dead cells. Similarly, higher levels of suspended solids, turbidity and inhibitory substances interfered with PCR or PMA-qPCR in water [76,77]. Interference to EMA-qPCR and PMA-qPCR may also be due to the presence of viable but nonculturable cells.

Contrary to PCR being widely reported as more sensitive than culturing, Pryor *et al.* [17] reported more consistent detection of *Legionella* spp. by culture methods compared to PCR; with more *Legionella* sp. isolated at 30 °C than the typical 35 °C incubation temperature. Semi-nested PCR was conducted with LEG 225 and LEG 858 primers enclosing 654 bp in the first step and with LEG 448 and LEG 858 in the second step. However, PCR was conducted on DNA from single presumed Legionella colonies but there is no indication as to how the colonies, which were PCR negative had been confirmed to be *Legionella* sp. in culture in the first place. Although acid treatment of samples prior to plating on BCYE had been conducted, this process only reduces but does not completely guard against growth of other organisms on the media.

### 3.1. Control of Legionella spp. and Protozoa in Recycled Water

A number of measures involving physical, thermal and chemical means are used to control *Legionella* sp. and protozoa in recycled water. Physical methods include use of filtration whereas thermal methods rely on freezing, heating and pasteurization techniques. Berk *et al.* [34] subjected samples to three cycles of freeze-thawing (−70 °C and +35 °C) followed by sonication to destroy amoeba trophozoites. The treatment left the structural integrity of their vesicles and embedded Legionella intact.

Most widely used by the industry to control *Legionella* sp. and protozoa are chemical disinfectants, particularly oxidizing agents such as chlorine, chlorine dioxide, chloramine, and ozone. Other oxidizing agents include iodine, hydrogen peroxide, potassium permanganate and bromine but these are rarely used and will not be discussed further. Because of its importance to the industry, photochemical disinfection using UV is also reviewed. The disinfectant should ideally be able to inactivate microorganisms in bulk water, control or remove biofilm and inactivate microorganisms associated with that biofilm. Overall, the efficacy of disinfectants depends on the culture condition of *Legionella* spp. and their host protozoa. For example, Cargill *et al.* [78] reported more susceptibility to chlorine and iodine to *Legionella* sp. grown on agar media than broth culture. Similarly, unattached (*i.e.*, plankatonic) *Legionella* sp. were hundredfold more susceptible to iodine than biofilm-based organisms which required more disinfectant to penetrate the biofilm. It is also more difficult to kill Legionella associated with protozoa and even more difficult when the bacteria are associated with cysts [79,80]. Specific disinfectants can also be impacted by chemical parameters such as organic matter content, pH and temperature as discussed for each disinfectant underneath. All of these considerations are important when designing a management strategy.

**Figure 4:**
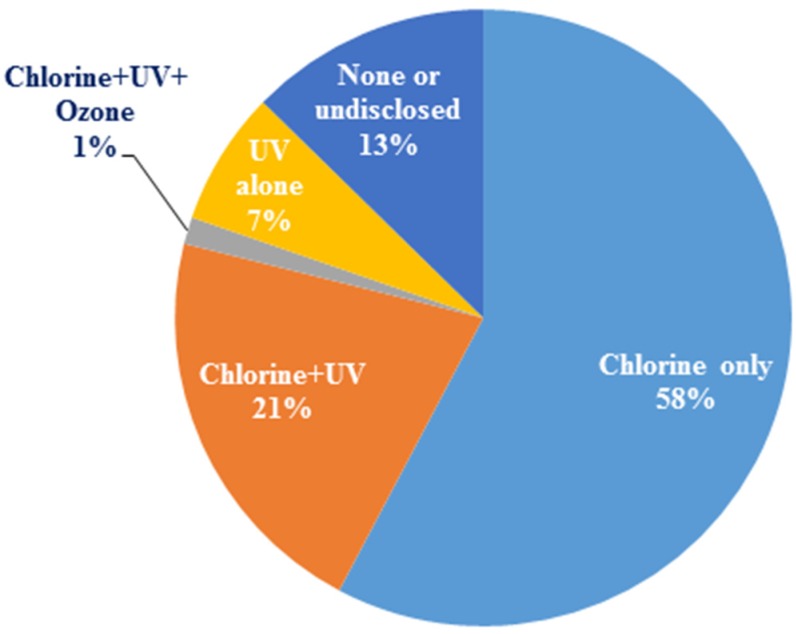
Disinfectants used by the recycled water industry (Note: Chlorine refers to all chlorine-based disinfectants as no distinction was made during the survey; Source: [25]).

In a recent survey of 71 recycled water plants in the US and Australia, chlorine was the dominant disinfectant, although a few utilities combined it with UV or ozone (Figure 4). Seven percent of the utilities used only UV disinfection, but more than three times as many utilities combined UV with another disinfectant, such as sodium hypochlorite, to provide a residual. A few (13%) other utilities did not disinfect or disclose information about disinfection practices. Choice of the disinfectant depended on cost, efficacy, ease of handling and preference. Disinfectant efficacy is often standardized based on the concept of a CT value (*i.e.*, concentration × time of exposure) necessary for a 2-log (CT_99%_) or 3-log (CT_99.9%_) inactivation. Because *Legionella* sp. can use protozoa as a protective shield against disinfectants, it is imperative to consider the efficacy of each disinfectant to both organisms. Efficacy of specific disinfectants on *Legionella* sp. and host protozoa is reviewed underneath.

### 3.2. Chlorine

Chlorine adversely affects the respiratory, transport activities and nucleic acids of microorganisms, leading to their inactivation [32]. Unlike potable water where trihalomethane concentrations of 80 μg/L or less are recommended by the USEPA, high chlorine residuals would be preferred to the point of use for some non-potable recycled water purposes. A main exception to this generalization is where the recycled water is intended for direct potable reuse. Mouchtouri *et al.* [43] disinfected Legionella-positive cooling towers by circulating water with 5 mg free chlorine/L for 5 h (*i.e.*, CT = 25 mg min/L). Systems with pH >8.0 received higher free chlorine dosages of 15 to 20 mg/L to achieve the required disinfection level. Disinfection was considered successful when samples showed <1 CFU/mL. Planktonic *Legionella* spp. resuspended in water were completely eliminated within 3 min by 2 mg·L^−1^ free chlorine derived from sodium hypochlorite [81]. By comparison, *Legionella* spp. in protozoa cysts survived 25-fold more chlorine disinfectant after 18 h [82].

**Figure 5:**
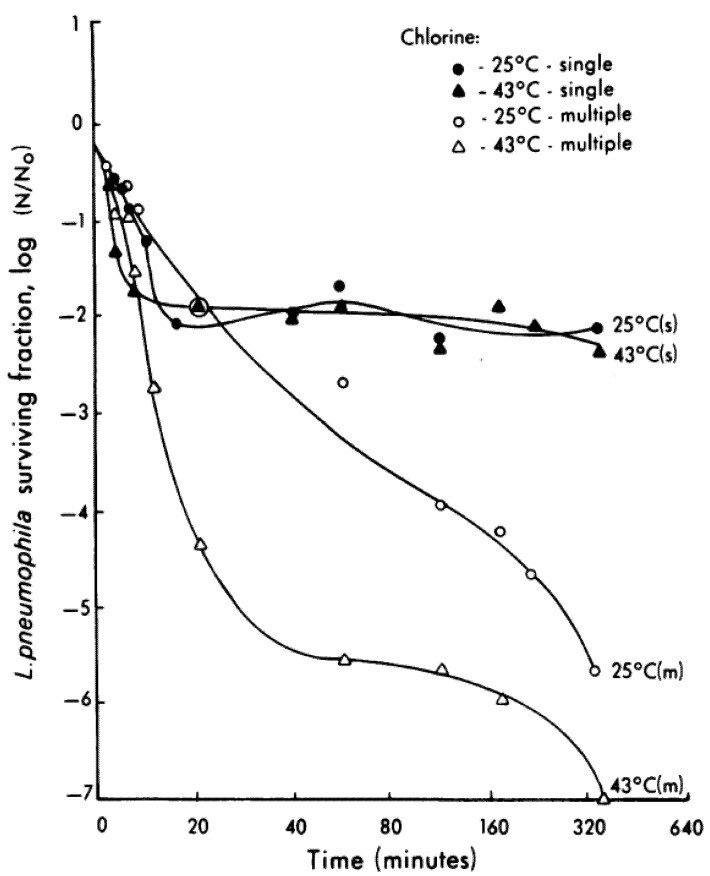
Efficacy of chlorine on *Legionella pneumophila* in a model system at ambient and high temperatures. To maintain a residual of 4 to 6 mg/L (attained with 18 mL at 25 °C and 40 mL at 43 °C), treatment with m received multiple application of chlorine whereas those with an s received a single dose of chlorine. (Source: [83] with permission from American Society for Microbiology).

Hyperchlorination with 4 to 6 mg/L decreased *L. pneumophila* in plumbing systems by 5 to 6 logs over 6 h [83]. The decline in *Legionella* sp. was more rapid at 43 °C than at 25 °C. However, a higher dose of chlorine was required at the higher temperature to overcome thermal decomposition and maintain a chlorine residual of 4 to 6 mg/L. The higher chlorine applications had to be applied in multiple doses as similarly high single applications were not effective over time (Figure 5). Chlorine was more effective with increasing temperatures implying that the warmer the water, the more efficacious is chlorine as a disinfectant. Enhanced efficacy of chlorine at higher temperatures is possibly due to accelerated binding of the chemical to the cell surface. This has practical applications, as it is easier to meet CT requirements in summer than in winter.

De Jonckheere and van de Voorde [84] documented more sensitivity to chlorine by *Naegleria* cysts (CT_99%_ of 9 to 30 mg min/L; pH 7.35 and 25 °C) as compared to *Acanthamoeba* cysts (CT_99%_ of 1260 to 6480 mg min/L; pH 7.35 and 25 °C; Table 5). *Acanthamoeba polyphaga* cysts required high levels of free chlorine (*i.e.*, 75 mg/L for a contact time of 18 h at 25 °C; a high CT of 81,000 mg min/L) to control excystation [82]. The pathogenic *Naegleria fowleri* were generally more susceptible to chlorine disinfection compared to non-pathogenic *N. gruberi*. However, some *Acanthamoeba* spp., another pathogenic organism and are fairly prevalent in recycled water, required a higher CT compared to *Naegleria* sp. and may require as much attention as *Naegleria* sp. in recycled water.

**Table 5:** Efficacy of chlorine on *Legionella pneumophila*, viruses and various protozoa.

Organism	Temp (°C)	pH	CT_99.9%_	Ref.
*Giardia lamblia*	25	7	41	[85]
*Giardia lamblia*	20	7	62	[85]
*Giardia lamblia*	15	7	83	[85]
*Giardia lamblia*	10	7	124	[85]
*Acanthamoeba* M3	30	8	12	[38]
*Acanthamoeba* M3 (infected with *Legionella* sp)	30	8	5	[38]
*Acanthamoeba* S2	30	8	37	[38]
*Acanthamoeba* S2 (infected with *Legionella* sp)	30	8	39	[38]
*Acanthamoeba* V1	30	8	70	[38]
*Acanthamoeba* V1 (infected with *Legionella* sp)	30	8	82	[38]
*Acanthamoeba* M3	50	8	5	[38]
*Acanthamoeba* M3 (infected with *Legionella* sp)	50	8	5	[38]
*Acanthamoeba* S2	50	8	5	[38]
*Acanthamoeba* S2 (infected with *Legionella* sp)	50	8	5	[38]
*Acanthamoeba* V1	50	8	28	[38]
*Acanthamoeba* V1 (infected with *Legionella* sp)	50	8	28	[38]
*Acanthamoeba* cysts	25	7.35	1260 to 6480 *	[84]
*Naegleria* cysts	25	7.35	9 to 30 *	[84]
*A. polyphaga* cysts	25	ND	81,000 *	[82]
Enterovirus	25	6–9	1	[85]
Enterovirus	20	6–9	2	[85]
Enterovirus	15	6–9	3	[85]
Enterovirus	10	6–9	4	[85]
*Legionella pneumophila*	25	ND	210	[83]
*Legionella pneumophila*	43	ND	60	[83]
*Legionella pneumophila serogroup* 1	ND	ND	9 *	[86]
*L. pneumophila*	30	8	4	[38]
*L. pneumophila* (in *Acanthamoeba* V1 co-culture)	30	8	38	[38]
*L. pneumophila* (in *Acanthamoeba* S2 co-culture)	30	8	44	[38]
*L. pneumophila* (in *Acanthamoeba* M3 co-culture)	30	8	50	[38]
*L. pneumophila*	50	8	3	[38]
*L. pneumophila* (in *Acanthamoeba* V1 co-culture)	50	8	3	[38]
*L. pneumophila* (in *Acanthamoeba* S2 co-culture)	50	8	3	[38]
*L. pneumophila* (in *Acanthamoeba* M3 co-culture)	50	8	3	[38]

* Only 2-log reduction (*i.e.*, CT_99%_).

Whereas *Legionella* sp. in environmental samples almost always occurs in the presence of host protozoa, only a few studies have looked at disinfectant efficacy in co-culture with protozoa. Dupuy *et al.* [38] used chlorine, chlorine dioxide and chloramine as disinfectants. Co-cultured amoeba and *L. pneumophila* were enumerated by initially centrifuging (14,000 g, 5 min) the sample and vortexing for 1 min to release intra-trophozoite bacteria. Aliquots of 1, 0.1, 0.01, and 0.001 mL were then plated on non-nutrient agar (NNA) with an *E. coli* lawn and on BCYE to enumerate protozoa and *Legionella* sp., respectively. NNA plates were incubated at 25 °C for 14 days (for amoeba) and BCYE plates were incubated at 37 °C for seven days (for *Legionella* sp.). Their results confirmed the superior efficacy of chlorine at higher compared to lower temperatures but also showed wide differences in efficacy if both *Legionella* sp. and amoeba are targeted (Table 5). Efficacy to chlorine disinfection between infected and non-infected *Acanthamoeba* sp. and/or *L. pneumophila* was negatively impacted, especially at the lower temperature (*i.e.*, 30 °C). Also included in Table 5 are CT values for *Giardia lamblia* and enterovirus as a point of reference for modeling risk (discussed later). Although both *Legionella* sp. and amoeba trophozoites have lower CTs than *G. lamblia*, higher CTs may be required to get rid of amoeba cysts using chlorine.

The high chlorine concentrations under hyperchlorination can corrode the pipes and appurtenances. Use of appurtenances with protective coating, such as sodium silicate and other anti-corrosion agents, has been proposed to reduce corrosion [32]. Other alternatives include the use of plastic-based infrastructure such as polyvinyl chloride.

Typical chlorine residuals in recycled water systems for ten systems surveyed recently in the US are presented in Table 6. The average chlorine residual was 0.3 mg/L and the median was 0.15 mg/L. A typical challenge is for utilities to maintain a chlorine disinfectant residual in the recycled water distribution system due to the inherently high organic carbon of the water which can sequester the disinfectant. Loret *et al.* [87] reported a significant positive correlation between free-living amoebae and dissolved organic matter. The chlorine also became increasingly ineffective in the distribution system as water temperature decreased the further the water flowed from the effluent point and reservoir (Table 6). Although the density of *Legionella* spp. decreased with distance from the effluent point in a number of cases, the possibility of most Legionella “hiding” in biofilms cannot be ruled out. Once in the biofilms, they become even more protected from the disinfectant and can be periodically released into the bulk water when the biofilm slough off.

An average pH 7.3 and median pH 7.6 was recorded in recycled water distribution systems (Table 6). In general, the lower the pH, the more efficacious the chlorine because chlorine exists in water as hypochlorous acid with a pKa of 7.6 which at pH < 7.6 is in a neutral form (*i.e.*, HOCl) whereas at pH > 7.6 exists as hypochlorite ion (OCl^−^). Disinfection with chlorine is impacted by pH as hypochlorite (OCl^−^) ions are less biocidal than the hypochlorous acid (*i.e.*, HOCl; [32,85]). This has operational management implication in cooling systems as they operate in a fairly alkaline range [42]. Recycled water pH in the reservoir and distribution system monitored for four consecutive days in Texas and Florida increased compared to the effluent [88]. Such increases can ultimately impact continued efficacy of the remaining residual downstream in the system.

Each system is very different in terms of length, total carbon and other parameters and this could affect the rate of chlorine decay. The chlorine data in Table 6 were used to determine chlorine decay in each system with distance, water temperature and TOC as independent variables (Table 7). Also presented was a summary of wastewater treatment technologies and pertinent practices which could impact disinfectant residuals in the distribution system. For CA-2 and CA-3, 87% of the chlorine dissipation (reflected by the coefficient of determination, R^2^) was explained by the system length and the decay rate was 0.071 and 0.051 mg Cl/mile, respectively. Water temperatures accounted for 87% and 94% of the chlorine decay in the FL-1 and NC system, respectively. Temperature also accounted for some of the disinfectant decay in the CA-1 and FL-5 systems, but only to a relatively small extent. TOC moderately accounted for decay in AZ-8 (61%), TX-3 (38%) and CO-5 (35%).

**Table 6:** Distance and physicochemical characteristic effects on chlorine residual and *Legionella* spp. occurrence in ten reclaimed water systems.

Site and Location ^a^		Distance (Miles)	TOC (mg/L)	pH	Temp (°C)	Free Cl (mg/L)	Legionella (CFU/mL)
CA-18	Effluent	0	5.0 ± 0.3	7.6 ± 0.1	20.5 ± 0.3	N/A ^b^	<3
	Reservoir	0.004	4.8 ± 0.1	7.7 ± 0.3	23.1 ± 0.3	0.1 ± 0	<3
	DS1	0.3	4.9 ± 0	7.8 ± 0.3	20.8 ± 0.2	0.1 ± 0	<3
	DS2	1.5	4.6 ± 0.1	8.0 ± 0	21.3 ± 0.2	0.1 ± 0	3
	DS3	2.5	4.7 ± 0.2	8.2 ± 0	19.6 ± 0.1	0.1 ± 0	46 ± 19
FL-1	Effluent	0	4.3 ± 0.1	7.7 ± 0.1	29.5 ± 0.1	2.03 ± 0.06	2300
	Reservoir	0.05	4.1 ± 0	7.8 ± 0	28.9 ± 0.4	1.17 ± 0.15	<3
	DS1	0.4	4.2 ± 0	7.8 ± 0	29.1 ± 0.2	0.55 ± 0.05	<3
	DS2	1.1	3.9 ± 0	7.8 ± 0	27.5 ± 0.2	0.12 ± 0.03	3
	DS3	2.7	3.8 ± 0	8.1 ± 0.1	27.0 ± 0.1	0.10 ± 0	29 ± 2
NC	Effluent	0	6.0 ± 0	7.0 ± 0	21.1 ± 0.5	0.65 ± 0.09	36
	Reservoir	7.9	5.7 ± 0.1	6.9 ± 0	16.3 ± 0.3	0.1 ± 0	55
	DS1	0.8	6.0 ± 0	7.2 ± 0.1	17.3 ± 0.4	0.2 ± 0.03	9
	DS2	8.0	6.1 ± 0.2	7.5 ± 0.1	16.4 ± 0.1	0.22 ± 0.03	47 ± 17
	DS3	9.3	6.0 ± 0	7.1 ± 0.1	15.8 ± 0.4	0.67 ± 0	23 ± 9
CA-3	Effluent	0	6.8 ± 0.1	8.2 ± 0	22.6 ± 1	1.33 ± 0.06	660 ± 300
	Reservoir	21.1	7.1 ± 0.1	7.6 ± 0	24.2 ± 0.3	0.16 ± 0.05	9
	DS1	11.5	7.1 ± 0.1	8.5 ± 0.1	23.4 ± 0.2	1 ± 0	140 ± 30
	DS2	17.9	7.1 ± 0.1	7.7 ± 0	24.7 ± 0.2	0.21 ± 0.04	<3
	DS3	26.9	6.7 ± 0	7.5 ± 0	23.3 ± 0.9	0.16 ± 0.02	6
CA-2	Effluent	0	6.1 ± 0.1	7.1 ± 0.1	23.5 ± 0.4	0.21 ± 0.04	<3
	Reservoir	0.002	7.1 ± 0.1	6.9 ± 0	29.6 ± 0.7	0.21± 0.02	1600 ± 1100
	DS1	0.63	6.4 ± 0.1	7.2 ± 0	27.2 ± 0.2	0.15 ± 0.03	24
	DS2	1.71	6.5 ± 0.1	7.1 ± 0	24.0 ± 0.5	0.14 ± 0.02	45
	DS3	2.62	6.4 ± 0.1	7.2 ± 0	21.3 ± 0.1	<0.01	3
CO-5	Effluent	0	7.5 ± 0.1	7.3 ± 0.1	23.6 ± 0.9	23.6 ± 0.9	130
	Reservoir	0.006	7.5 ± 0.1	9.5 ± 0	27.8 ± 0.8	0.15 ± 0.03	460 ± 160
	DS1	0.1	7.5 ± 0.1	7.3 ± 0	22.4 ± 0.9	0.14 ± 0.02	810 ± 340
	DS2	1.3	6.3 ± 0	7.8 ± 0.1	23.6 ± 0.4	0.11 ± 0.01	220 ± 270
	DS3	2	7.4 ± 0.1	7.3 ± 0	22.7 ± 1.0	0.18 ± 0.02	92 ± 2
CA-1	Effluent	0	4.9 ± 0	8.1 ± 0	26.7 ± 0.5	0.16 ± 0.04	<3
	Reservoir	0.1	5.7 ± 0	8.6 ± 0	19.9 ± 0.2	<0.01	220 ± 270
	DS1	2.3	8.1 ± 0.1	7.4 ± 0	21.0 ± 0.8	0.03 ± 0.03	3300 ± 1900
	DS2	4.4	6.8 ± 0.1	7.6 ± 0	20.2 ± 0.6	0.14 ± 0.02	45
	DS3	6.9	7.4 ± 0	7.7 ± 0	22.6 ± 0.2	0.13 ± 0.02	12
AZ-8	Effluent	0	6.1 ± 0.1	7.9 ± 0	15.2 ± 1.2	0.22 ± 0.03	<3
	Reservoir	0.9	2.4 ± 0	8.4 ± 0	17.0 ± 0.2	0.11 ± 0.04	12
	DS1	0.1	1.7 ± 0	8.4 ± 0	13.9 ± 0.6	0.04 ± 0.02	30
	DS2	1.2	1.7 ± 0	8.0 ± 0	13.8 ± 0.6	0.14 ± 0.02	3
	DS3	2	2.0 ± 0	7.9 ± 0	13.5 ± 0.8	0.05 ± 0	<3
TX-3	Effluent	0	7.5 ± 0.1	7.8 ± 0.2	22.9 ± 0.1	0.4 ± 0.3	120 ± 130
	Reservoir	1.5	7.7 ± 0.1	7.4 ± 0.1	21.9 ± 1	0.3 ± 0.1	500 ± 310
	DS1	1.9	6.4 ± 0.1	7.3 ± 0.1	22.5 ± 0.1	0.08 ± 0.03	3
	DS2	4.9	5.5 ± 0.1	7.3 ± 0.1	22.1 ± 0.1	<0.01	3
	DS3	6.4	4.9 ± 0.1	7.9 ± 0	23.0 ± 0.3	0.06 ± 0.1	9
FL-5	Effluent	0	13.9 ± 0.2	7.4 ± 0	28.3 ± 0.1	1.53 ± 0.06	870 ± 990
	Reservoir	0.04	15.0 ± 0	7.5 ± 0	26.9 ± 0.5	0.48 ± 0.08	45 ± 19
	DS1	0.5	14.2 ± 0.1	7.7 ± 0	24.7 ± 0.2	0.14 ± 0.05	33
	DS2	3.3	8.4 ± 0.1	7.2 ± 0	26.2 ± 0.2	0.08 ±0.03	105 ± 140
	DS3	6.8	8.4 ± 0.1	7.1 ± 0	27.4 ± 0.3	0.13 ± 0.03	45
Mean		6.8 ^c^	6.3	7.3	22.7	0.30	440
Median		4.6 ^c^	6.2	7.6	22.9	0.15	94

^a^ Sampled in March (CA-18 and TX-3), April (FL-1, FL-5 and NC), May (AZ-8, CA-1, CA-2, CA-3) or July (CO-5) of 2013; ^b^ Not applicable (No chlorination); ^c^ Mean and median of total mile length of each system as opposed to the mean and median of respective sampling points. (Source: [25]).

### 3.3. Chlorine Dioxide

Chlorine dioxide (ClO_2_) is another oxidizing disinfectant of increased importance in recycled water. However, it decomposes readily and presents storage challenges. Thus, where used, it is typically generated onsite for immediate use by slowly adding a strong acid (e.g., sulfuric acid) to sodium chloride solution. Chlorine dioxide impacts microorganisms by disrupting protein synthesis. Walker *et al.* [89] reported total elimination of *Legionella* sp. in a hospital water system after treatment with 50–80 mg/L chlorine dioxide. Its efficacy on *Legionella* sp. and *Acanthamoeba* sp. in comparison to *Giardia* sp. and enterovirus is presented in Table 8. Based on those results, it is more potent than chlorine. It also shows better efficacy on biofilms compared to chlorine [32,89]. Unlike chlorine, its efficacy is less dependent on pH changes, but, just like chlorine, it is affected by temperature (Table 8).

**Table 7:** Chlorine decay in ten recycled water systems.

Site	System Practices ^a^	Characteristic	Decay Model	R^2^	Decay Rate
CA-18	Plant with 350 MG/yr capacity using MBR followed by UV disinfection. System with drip irrigation. Water has 1 day shelf-life in the distribution system.	Distance	y = 2E − 17x + 0.1	0	0 (No decay)
		Temperature	y = 3E − 16x + 0.1	0	
		TOC	y = 7E − 15x + 0.1	0	
FL-1	20 MGD sewage plant with 5-stage biological nutrient removal (BNR; *i.e.*, Bardenpho system) with enhanced removal of nitrogen and phosphorus. System was well pressurized, and the water was used rapidly (*i.e.*, within 1–3 days).	Distance	y = 1.0424e^−1.04x^	0.739	1.16 mg Cl/°C
		Temperature	y = 2E − 15e^1.1629x^	0.870	
		TOC	y = 8E − 13e^6.6594x^	0.837	
NC	50 MG/yr using AS technology. Effluent disinfected with UV and chlorine. Booster disinfection at the furthest point sampled ( *i.e.*, DS3; not included in the decay model).	Distance	y = 0.4176e^0.134x^	0.576	0.104 mg/°C
		Temperature	y = 0.104x − 1.5489	0.940	
		TOC	y = 1E − 0.8e^2.8477x^	0.438	
CA-3	110 MGD wastewater plant by AS, tertiary treatment and dual media (anthracite/sand) filtration. System had multiple pressure zones (40–200 psi).	Distance	y = −0.0505x + 1.3543	0.872	0.051 mg Cl/mile
		Temperature	y = 0.3513x + 8.879	0.387	
		TOC	y = −0.2615x + 2.3947	0.008	
CA-2	Facultative ponds (lagoons; 3 mg DO/L) followed by multiple ponds with aerators to attain 8–10 mg DO/L). Water subjected to DAF (at 70 to 80 psi), creating microbubbles. System branched but without any dead ends. Reservoir is aerated. Water used within 2 days or discharged into river.	Distance	y = −0.0695x + 0.2117	0.8126	0.07 mg Cl/mile
		Temperature	y = 0.0176x − 0.2998	0.424	
		TOC	y = 0.0383x − 0.1065	0.027	
CO-5	40 MG/yr AS with extended aeration (DO to approximately 1 mg/L to drive nitrification. UV disinfection (fluence of 40,635.28 mJ/cm^2^) and chlorine gas (10 to 15.l b gas/day in 1000 gal/min.) then filtered through a pack of eight cloth filters. Reservoir was aerated.	Distance	y = 0.0087x + 0.1387	0.073	0.22 mg Cl/mg TOC (R^2^ low due to other characteristics e.g., heavy algal growth in reservoir)
		Temperature	y = 0.0011x + 0.1718	0.008	
		TOC	y = −0.0287e^0.2211x^	0.352	
CA-1	16 MGD AS process with an anoxic phase to facilitate nitrification combined with a fine bubble diffuser. Clarified liquid was filtered through thick anthracite and coal filtration beds. The filtered water was disinfected with UV (fluence of 144,000–180,000 mJ/cm^2^) before chlorination.	Distance	y = 0.0095x + 0.0659	0.145	0.015 mg Cl/°C (R^2^ low due to other characteristics e.g., multiple pressure zones with 14 to 90 psi)
		Temperature	y = 0.0153x − 0.2456	0.337	
		TOC	y = −0.0119x + 0.1703	0.043	
AZ-8	Production capacity of 0.28 MGD. The aeration tanks had an anoxic zone where the mixed liquor dissolved solids attained a low DO (0.07 mg DO/L). Disinfection was achieved with chlorine gas followed by gravity-fed filtration (sand and anthracite). Distributed through a looped system.	Distance	y = −0.0353x + 0.1411	0.151	0.031 mg Cl/mg TOC (R^2^ low due to other characteristics e.g., disinfectant retention by filters)
		Temperature	y = 0.0056e^0.1894x^	0.157	
		TOC	y = 0.0313x + 0.0249	0.609	
TX-3	60 MGD plant with an activated sludge process. System had some dead ends. The dissolved oxygen was greatly diminished in the distribution system as well and the water was rusty due to corrosion.	Distance	y = 0.3058e^−0.745x^	0.336	1.69 mg Cl/mg TOC (R^2^ low due to other factors e.g., corrosion)
		Temperature	y = 2E − 15e^1.3472x^	0.069	
		TOC	y = 7E − 07e^1.6884x^	0.377	
FL-5	The anaerobic-anoxic-oxic (*i.e.*, A2O) plant had a 2.75 MGD capacity. The treatment process removed BOD and TSS as well as reduced nitrogen and phosphorus.	Distance	y = 0.4263e^−0.246x^	0.347	0.304 mg Cl/°C (R^2^ low due to other characteristics e.g., multiple dead ends and pressure zones)
		Temperature	y = 0.3041x − 7.6502	0.457	
		TOC	y = 0.137e^0.2437x^	0.421	

^a^ AS = Activated sludge; BNR = biological nutrient removal; MG = Million gallons; MBR = membrane bioreactor; DAF = dissolved air floatation; DO = dissolved oxygen.

**Table 8:** Efficacy of chlorine dioxide on *Legionella pneumophila*, viruses and various protozoa.

Organism	Temp (°C)	pH	CT _99.9%_	Ref.
*Giardia lamblia*	25	6–9	11	[85]
*Giardia lamblia*	20	6–9	15	[85]
*Giardia lamblia*	15	6–9	19	[85]
*Giardia lamblia*	10	6–9	23	[85]
*Acanthamoeba* M3	30	8	0.5	[38]
*Acanthamoeba* M3 (infected with *Legionella* sp)	30	8	0.5	[38]
*Acanthamoeba* S2	30	8	2.1 *	[38]
*Acanthamoeba* S2 (infected with *Legionella* sp)	30	8	5.5 *	[38]
*Acanthamoeba* V1	30	8	0.4 *	[38]
*Acanthamoeba* V1 (infected with *Legionella* sp)	30	8	3.5 *	[38]
*Vermamoeba vermiformis*	20	7.6–7.8	300 *	[90]
Enterovirus	25	6–9	-	[85]
Enterovirus	20	6–9	6.4	[85]
Enterovirus	15	6–9	8.6	[85]
Enterovirus	10	6–9	12.8	[85]
*Legionella* sp.	ND	ND	0.08	[91]
*Legionella pneumophila*	30	8	0.4	[38]
*L. pneumophila* (in *Acanthamoeba* V1 co-culture)	30	8	2.8	[38]
*L. pneumophila* (in *Acanthamoeba* S2 co-culture)	30	8	0.9 **	[38]
*L. pneumophila* (in *Acanthamoeba* M3 co-culture)	30	8	2.4	[38]

* Only 1 log reduction (*i.e.*, CT_90%_); ** Only 2-log reduction (*i.e.*, CT_99%_).

### 3.4. Monochloramine

From a practical perspective, monochloramine can be locally generated by adding free chlorine in a solution of ammonium chloride at a chlorine to nitrogen molar ratio of 0.5 (pH 8.5). Also formed during the process are dichloramine and nitrogen trichloramine. However, monochloramine is generally most predominant of the three at neutral pH or higher [32]. The three products are commonly referred to as “combined” chlorine. Disinfection with chloramine gained traction in the US because the disinfectant is more stable in the system, minimizes the formation of disinfection by-products, and can penetrate biofilms better compared to free chlorine [15]. Its efficacy against *Legionella* sp. was demonstrated in various systems [15,92]. In a different study, monochloramine concentrations of 1–4 mg/L as Cl_2_ significantly reduced the occurrence of *Legionella* sp. in a hospital water system [93]. The wide range of monochloramine concentrations required was possibly due to pH as disinfection with chloramine requires an optimal pH of approximately 7.5. Its use led to a less diverse *Legionella* spp. population in the distribution system of water with a high average TOC content of 4 mg/L [17]; typical of recycled water. The occurrence of *Legionella* sp. in showerheads and cooling towers on switching from chlorine to chloramine decreased from 20% to 6.2% although the density of *L. pneumophila* (detected via 16S rRNA and direct culturing) remained the same, suggesting resistance of this species to chloramine.

From an epidemiologic perspective, US hospitals supplied with water disinfected with chlorine were more likely to have reported outbreak of Legionnaires’ disease than hospitals that used monochloramine as a disinfectant (odds ratio 10.2 [95% confidence interval 1.4-460]; Kool *et al.*, [15]). This implied that hospitals supplied with water containing free chlorine were 10.2 times more likely to experience a Legionnaires’ disease outbreak. However, that study was entirely based on infections and not substantiated by field data on the occurrence of *Legionella* sp. in the studied areas.

The efficacy of chloramine to *Legionella* sp. and amoeba in comparison to *Giardia lamblia* and enteroviruses is summarized in Table 9. Those data show much lower CTs for *Legionella* sp. and amoeba compared to *G. lamblia* and enteroviruses even in instances where the *Legionella* sp. are embedded in amoeba.

**Table 9:** Efficacy of chloramine on *Legionella pneumophila*, viruses and various protozoa.

Organism	Temp (°C)	pH	CT _99.9%_	Ref.
*Giardia lamblia*	25	6–9	750	[85]
*Giardia lamblia*	20	6–9	1100	[85]
*Giardia lamblia*	15	6–9	1500	[85]
*Giardia lamblia*	10	6–9	1850	[85]
*Acanthamoeba* M3	30	8	19	[38]
*Acanthamoeba* M3 (infected with *Legionella* sp)	30	8	20	[38]
*Acanthamoeba* S2	30	8	40 *	[38]
*Acanthamoeba* S2 (infected with *Legionella* sp)	30	8	47 *	[38]
*Acanthamoeba* V1	30	8	23	[38]
*Acanthamoeba* V1 (infected with *Legionella* sp)	30	8	24	[38]
Enterovirus	25	6–9	356	[85]
Enterovirus	20	6–9	534	[85]
Enterovirus	15	6–9	712	[85]
Enterovirus	10	6–9	1067	[85]
*Legionella pneumophila*	30	8	17	[38]
*L. pneumophila* (in *Acanthamoeba* V1 co-culture)	30	8	23	[38]
*L. pneumophila* (in *Acanthamoeba* S2 co-culture)	30	8	22	[38]
*L. pneumophila* (in *Acanthamoeba* M3 co-culture)	30	8	19	[38]

* CT 99% data.

### 3.5. Ozone

Ozone has been used to inactivate microorganisms in recycled water for almost three decades [94,95,96]. Ozone attacks unsaturated bonds of aldehydes, ketones, and carbonyl compounds [97] and can participate in electrophilic reactions with aromatic compounds and neutrophilic reactions with many cellular components (*i.e.*, fatty acids, carbohydrates, amino acids, proteins, and nucleic acids). These reactions collectively affect the cytoplasmic membrane of bacterial cells and the protein structure as well as DNA. However, because it does not form a stable residual, it decomposes rapidly in the water. Thus, it is typically used by the recycled water industry in combination with other disinfectants (Figure 4).

Muraca *et al.* [83] provided 0.5 mg ozone/L, reducing *L. pneumophila* in 5 h by 5 log units from an initial concentration of 10^7^ cfu/mL (Table 10). Ozone efficacy was not impacted by temperature (25 °C *versus* 43 °C) or turbidity although the level of turbidity was not quantified. Temperatures above 30 °C occur rarely in US recycled water distribution systems (Table 6) but can be encountered in cooling towers. Based on Domingue *et al.* [86], the efficacy of ozone was not greatly affected by pH or temperature although others reported better efficacy against coliphage and bacteria at lower temperatures [98]. CT values from Muraca *et al.* [83] for reducing *Legionella* sp. were much higher than those reported by others possibly because that research group dosed their system as to maintain an ozone residual of 1 to 2 mg/L.

Much lower CT values are required to control both *Naegleria* and *Acanthamoeba* cysts with ozone at 25 °C although slightly high CTs may be required at lower temperatures of 20–22 °C (Table 10). Overall, ozone is more effective than chlorine dioxide, which was in turn more effective than chlorine (*i.e.*, O_3_ > ClO_2_ > Cl_2_); an observation that is in agreement with Miller and Simpson [42]. However, since ozone dissipates from the water much more quickly, it should preferably be used in combination with chlorine or chloramine to serve as a preservative.

**Table 10:** Efficacy of ozone on *Legionella pneumophila*, viruses and various protozoa.

Organism	Temp (°C)	pH	CT 99% (mg min/L)	Ref.
*Giardia lamblia*	25	6–9	0.48 *	[85]
*Giardia lamblia*	20	6–9	0.72 *	[85]
*Giardia lamblia*	15	6–9	0.95 *	[85]
*Giardia lamblia*	10	6–9	1.4 *	[85]
*Naegleria gruberi* (NEG)	25	7	1.3	[99]
*Naegleria gruberi* (NEG)	25	7	<1.6	[100]
*Naegleria gruberi* (1518/1d)	25	7	1.6	[100]
*Naegleria gruberi* (Echirolles)	25	7	<1.6	[100]
*Naegleria* spp. (MO5; C110; An24)	25	7	<1.6	[100]
*Naegleria fowleri*	25	7	<1.6	[100]
*Acanthamoeba polyphaga* (1501/3a)	25	7	2.5	[100]
*Acanthamoeba polyphaga*	20–22	7.5–8	5	[101]
*Acanthamoeba culbertsoni* (A1)	25	7	<1.6	[100]
*Acanthamoeba royreba* (OR)	25	7	<1.6	[100]
*Acanthamoeba* spp. (MR4)	25	7	1.6	[100]
*Hartmannella vermiformis*	25	ND	<1.6	[100]
Enterovirus	25	6–9	0.15	[85]
Enterovirus	20	6–9	0.25	[85]
Enterovirus	15	6–9	0.3	[85]
Enterovirus	10	6–9	0.5	[85]
*Legionella pneumophila*	25	ND	60	[83]
*Legionella pneumophila*	43	ND	55	[83]
*Legionella pneumophila* serogroup 1	25–45	7.2	0.5	[86]
*Legionella pneumophila* serogroup 1	25	8	0.95	[86]
*Legionella pneumophila* serogroup 1	25	8.9	0.65 *	[86]

ND: Not determined; * CT_99.9_ data.

### 3.6. UV

UV does not kill microorganisms but rather damage their DNA, which prevents them from reproducing. Preventing reproduction in turn prevents infectivity. Similar to the CT concept, UV intensity (mW-s/cm^2^) times the exposure time(s) commonly referred to as fluence (mJ/cm^2^) describes UV disinfection capability. Fluence represents the energy per unit area falling onto a surface. Maximum efficacy with UV is attained at 254 nm [32] but turbidity, natural organic matter content and particulate matter can significantly affect UV disinfection capability. UV irradiation at 30 mJ/cm^2^ reduced *L. pneumophila* by 5 log units in 20 min ([83]; Table 11). Continued exposure to this dose for 6 h still left a residual of 10^2^ cfu/mL. Schwartz *et al.* [102] detected *Legionella* sp. in biofilms formed on polyethylene, polyvinyl chloride (PVC), and stainless steel coupons following disinfection with UV but no *Legionella* sp. was detected on copper coupons. UV disinfection was not affected by temperature. UV efficacy was also independent of pH [103]. *Legionella* sp. was inactivated within 3 min on exposure to ultraviolet light at 90 mJ/cm^2^ [81]. All Legionella isolates tested by Cervero-Aragó *et al.* [104] required 5–6 mJ/cm^2^ UV fluence to inactive 4 logs. However, a higher fluence was required when co-cultured with amoeba (Table 11).

**Table 11:** Efficacy of UV on *Legionella pneumophila*, in comparison with Giardia and enterovirus.

Organism	Fluency (mJ/cm^2^) for Respective Inactivation				Ref.
	1 Log	2 Logs	3 Logs	4 Logs	
*Giardia*			11		[103]
*Giardia*			20–80		[105]
*Giardia*		<10			[105]
*Acanthamoeba* sp.	40				[106]
*A. castellani* CCAP 1534/2 (Trophozoites)	32.1		22.7		[104]
*A. castellani* CCAP 1534/2 (Cysts)	45.4		90.9		[104]
*Acathamoeba* sp. 155 (Trophozoites)	27.6		65.7		[104]
*Acathamoeba* sp. 155 (Cysts)	34.2		99.2		[104]
*V. vermiformis* CCAP 1534/7A (Trophozoites)	10.7		26		[104]
*V. vermiformis* CCAP 1534/7A (Cysts)	16.8		53.8		[104]
*V. vermiformis* 195 (Trophozoites)	10.1		24.2		[104]
*V. vermiformis* 195 (Cysts)	31.5		76.2		[104]
Enterovirus				186	[103]
*Legionella pneumophila* sg. 1 ATCC 33152	1.7			5.7	[104]
*Legionella pneumophila* sg. 1 env ^a^	1.7			5	[104]
*Legionella pneumophila* sg. 7 ATCC 33823	1.7			5	[104]
*Legionella pneumophila* sg. 8 env ^a^.	1.8			6.1	[104]
*Legionella pneumophila* ATCC 33462	1.4			6.3	[104]
*Legionella pneumophila* sg. 1 env			4		[104]
*Legionella pneumophila* sg. 1 env with *A. castellani* CCAP 1534/2			6		[104]
*Legionella pneumophila* sg. 1 env with *Acathamoeba* sp. 155			8		[104]
*Legionella pneumophila* (25 °C and 43 °C)			30		[83]

a *L. pneumophila* sg. 1 env and *L. pneumophila* sg. 8 env were environmental isolates.

Hijnen *et al*. [106] reported a log reduction of *Acanthamoeba* sp. with 40 mJ/cm^2^, a fluence sufficient for impacting adenoviruses as well. Three log units of various *Acanthamoeba* species and *V. vermifomis* were inactivated with 23 to 100 mJ/cm^2^ UV, the higher fluence being required for cyst inactivation (Table 11). Overall, inactivation of *Acanthamoeba* sp. and *Vermamoeba veriformis* required higher levels of UV compared to *Giardia* sp. Generally, UV light is most effective on protozoa followed by bacteria and least effective against viruses. However, this generalization does not seem to apply to *Legionella* sp. as high fluence was required by Muraca *et al.* [83] for any impact (Table 11). Because UV does not provide a residual, it is typically combined with a chemical disinfectant ion for effectively controlling *Legionella* sp.

### 3.7. Copper-Silver Ionization

Both copper and silver have biocidal activity. In ionization chambers, both metals can be ionized through electrolysis forming positively charged ions of each metal. The copper ions form electrostatic compounds with negatively charged cell walls of *Legionella* sp. (and other bacteria), disrupting cell wall permeability and subsequent nutrient uptake. The ions penetrate the cell wall and create an entrance for silver ions (Ag^+^) which penetrate the cells and bond with DNA, RNA, cellular proteins and respiratory enzymes, immobilizing the cell and curtailing cell division. This chain of events leads to death. Thus, combining both metal ions has a synergistic effect. Copper-silver ionization has been widely used to control *Legionella* sp. in various settings [22]. The technology was quite effective against *Legionella* sp. at copper-silver ionization concentrations of 0.36/0.04 mg/L but slightly higher concentrations of 0.4/0.04 may be required in large systems. The metals also effectively penetrated the biofilm. However, Cu-Ag ionization efficacy can be impacted by water pH and TDS. With pH9, only one tenth of all *Legionella* sp. were eliminated. The silver will precipitate in the presence of high dissolved solid concentrations becoming unavailable for disinfection. Most studies have looked at the disinfection effects of these ions used together but Lin *et al.* [107] examined the effects of each ion individually. They reported complete inactivation of *L. pneumophila* serotype 1 in 2.5 h (6 log reduction) with 0.1 mg/L copper. Similarly, *L. pneumophila* was killed within 6 h on exposure to a solution of 50 μg/L silver ions [80].

### 3.8. Other Disinfecting Agents

Bromine as a disinfectant behaves in a similar fashion as chlorine, existing in water as hypobromous acid (HOBr) and hypobromite ion (OBr^−^) depending on the pH [32]. At neutral pH, HOBr is the predominant species (pKa of 8.8 which is a unit higher than chlorine). Bromine has generally less efficacy against *Legionella* sp. compared to chlorine. Bromine at CT = 576 to 1440 mg min/L, iodine at CT = 2880 to 7200 mg min/L, and iodophore at CT = 2880 to 7200 mg min/L) were ineffective against *Acanthamoeba culbertsoni* cysts at pH 7.5 [84]. By contrast, these CT values with iodine and ionophore had acceptable cysticidal effect on *Neagleria fowleri*. Although used for potable water disinfection in some instances, use of bromine, iodine and hydrogen peroxide in recycled water systems has not been documented. Other disinfectants and their related efficacy to *Legionella* sp. and protozoa are summarized in Table 10. Miller and Simpson [42] reaffirmed the resistant nature of protozoa cysts to disinfection with some of these disinfectants as well.

Like hydrogen peroxide, peracetic acid (CH_3_COOOH) is another peroxygen compound but with even higher potency that has not yet been exploited by the recycled water industry. It is thought to disinfect by impacting lipoproteins in the cell membrane [108]. Unlike chlorine and hydrogen peroxide, its potency is not greatly compromised by presence of organic matter or enzymes, respectively [109]. Although most effective against fecal indicator bacteria and viruses under mildly acidic conditions, it showed acceptable potency at neutral pH as well. It was also more effective against biofilms [108]. Peracetic acid has not been used to control *Legionella* sp. but was used against *Acanthamoeba* sp. and *Naegleria* sp. at different concentrations (Table 12). Its efficacy was more elevated in a combination of 0.2% PAA and 80% ethanol [110].

**Table 12:** Minimum lethal concentration of various biocides on protozoa.

Biocide	Minimum Lethal Dose (MLC; mg/L)							
	Acanthamoeba		Naegleria		Colpoda		Tetrahymena	Vannella
	Trophozoite	Cyst	Trophozoite	Cyst	Trophozoite	Cyst		
Peracetic acid ^a^	15	150	8	8	ND	ND	ND	ND
Chlorinated phenolic thioether ^a^	10	80	2	20	ND	ND	ND	ND
Isothiazolin ^a^	2	150	<1	2	ND	ND	ND	ND
Isothiazolin ^b^	244	31,250	ND	ND	31	7813	31	122
Polyhexamethylene biguanide ^a^	10	5	20	2000	ND	ND	ND	ND
Bromonitropropanediol ^a^	200	>10,000	50	25	ND	ND	ND	ND
Methylenebis thiocyanate ^a^	3	>1000	5	<1	ND	ND	ND	ND
Thiocarbamate ^b^	3906	125,000	ND	ND	977	31,250	244	3906
Quaternary ammonium compounds (QAC) ^b^	61	62,500	ND	ND	61	488	122	61
Tributyltin neodecanoate (TBT)/QAC ^b^	31	122	ND	ND	15	31	15	61
Chlorine ^a^	2	>50	2	4	ND	ND	ND	ND

References ^a^ [111] and ^b^ [42].

Berk *et al.* [34] tested Microbiocides MBC-115 and MBC-215 widely used in cooling towers to control *Legionella* spp. at final concentrations of 15 ppm (vol/vol) and 100 ppm (vol/vol), respectively to control amoeba. MBC-115 is a quaternary ammonium comprised of poly[oxyethylene (dimethyliminio)ethylene (dimethyliminio)ethylene dichloride (Nash-Chem, Nashville, TN). Its efficacy on *Legionella* spp. was dismal. MBC-215 is an isothiazine derivative of a mixture of 5-chloro-2-methyl-4-isothiazolon-3-one and 2-methyl-4-isothiazolin (Nash-Chem, Nashville, TN, USA). The concentration used was also ineffective on *Legionella* spp. However, efficacy of these microbiocides against *Legionella* spp. may be impacted by the conditions under which the target organism is growing. For example, polyhexamethylene bioguanide and benzisothiazolone were ineffective against *L. pneumophila* grown with *A. polyphaga* compared to *L. pneumophila* pure cultures [112]. Both microbiocides act by impacting the integrity of the bacteria cell membrane. The presence of amoebal proteins coating Legionella seems to confer biocide resistance.

Iron is a fundamental requirement for *Legionella* spp. but these organisms lack siderophores which are capable of competing with iron chelators. Thus, addition of lactoferrin, an iron chelator sequestered this essential nutrient, killing *L. pneumophila* [113]. The economic feasibility of this strategy to control *Legionella* sp. in full-scale recycled water treatment systems is unknown. On the opposite extreme, excessive amounts of iron inhibited biofilm formation [114]. Raftery *et al.* [115] documented reduced formation of *L. pneumophila* biofilms with nanoparticles. Interaction between *L. pneumophila* and amoeba in the presence of gold nanoparticels was also negatively impacted. These metal and nanoparticle considerations have not yet been fully explored as possible management strategy for *Legionella* sp. and protozoa in recycled water.

## 4. Summary and Conclusions

Whereas *Legionella* sp. in environmental samples almost always occurs in the presence of host protozoa, only a few studies have looked at disinfectant efficacy in co-culture with protozoa. Utilities typically face challenges in maintaining a disinfectant residual in the recycled water distribution system due to the inherently high organic carbon of the water, which can sequester the disinfectant. Furthermore, some Legionella strains may be inherently resistant to common disinfectants such as chloramine. Overall, use of a combination of disinfectants, e.g., UV combined with chlorination or ozone combined with chlorine is more likely to produce a more acceptable product. Some emerging disinfectants such as peracetic acid appear less prone to dissipation in the presence of organic matter. More research is needed to establish their efficacy in recycled water and the associated economics of use in full-scale systems. Legionella outbreaks tend to occur sporadically and in most instances the source of infection is not always easily deciphered. Although no outbreak has been associated with recycled water, the industry needs to proactively prevent future outbreaks. Studies to understand the role played by protozoa in establishing the infectious dose of *Legionella* spp. to humans need to be conducted.
